# Interspecies differences in membrane-associated protease activities of thyrocytes and their relevance for thyroid cancer studies

**DOI:** 10.1186/1756-9966-31-45

**Published:** 2012-05-16

**Authors:** Eleonore Fröhlich, Elke Maier, Richard Wahl

**Affiliations:** 1 Department of Endocrinology, Metabolism, Nephrology and Clinical Chemistry, Internal Medicine, University of Tuebingen, Otfried-Muellerstrasse 10, 72076, Tuebingen, Germany; 2 Institute of Anatomy, University of Tuebingen, Oesterbergstr 3, 72074, Tuebingen, Germany; 3 Center for Medical Research, Medical University of Graz, Stiftingtalstr 24, Graz, 8010, Austria

**Keywords:** Thyroid cancer, Protease activity, Dipeptidyl peptidase IV, Aminopeptidase N

## Abstract

**Background:**

To understand the role of proteases involved in human thyroid cancer progression and tissue invasion, thyrocytes from other species could potentially be used provided their characteristics are similar. It is not known whether dipeptidyl peptidase IV and aminopeptidase N activities, which are overexpressed in human thyroid cancer, are, as in human, also absent in normal thyrocytes of other species, making them suitable models for studies on the regulation of these proteases.

**Methods:**

To assess the role of these proteases, activity was measured in thyroid tissue of human, mouse, rat, porcine, bovine and ovine origin. The lysosomal protease, dipeptidyl peptidase II, was used for comparison.

**Results:**

Murine, rat, ovine, bovine and human thyrocytes all lacked dipeptidyl peptidase IV and aminopeptidase N activity, but porcine thyrocytes were found to possess both. In contrast, lysosomal dipeptidyl peptidase II was strongly expressed in all species. These activity patterns were maintained in cultured cells. Cultured porcine thyrocytes formed follicles with typical morphology upon stimulation with TSH but differed from human thyrocytes in their response to thiamazole.

**Conclusions:**

These species differences in the expression of dipeptidyl peptidase IV and aminopeptidase N, indicate that porcine thyrocytes cannot be considered appropriate for the study of proteases in human cancer development.

## Background

Normal thyrocytes are used for investigations of hormone synthesis, regulation of proliferation and differentiation and as controls in drug screening. Primary cells and cell lines of canine, porcine, bovine, ovine and rat origin are used to address different questions. Rat cell lines, especially the FRTL5 line, are used for proliferation studies [1], whereas porcine and bovine cells are used most commonly for differentiation and gene expression studies. Similar to ovine thyrocytes, cells from these species show a poor response to TSH and, therefore, are not suited for studies of proliferation [2]. Due to their limited availability, very few groups use canine thyrocytes for their studies. Despite conserved physiology, marked differences between these species have already been reported [3,4]. Stimulation with TSH and insulin triggers DNA synthesis in dog thyrocytes and rat cell lines by very different mechanisms.

Interspecies differences in the regulation of protease activities are of particular importance because several lysosomal and membrane-associated proteases promote tumor development and progression. The lysosomal enzymes cathepsin B and cathepsin L are over-expressed in thyroid cancer as in most other cancers [5,6]. Similar to other cancers, the participation of metalloproteinases, especially metalloproteinases (MMP) MMP-2, also termed type IV collagenase, in thyroid cancer progression has also been confirmed [7-9]. Additionally, the urokinase-type plasminogen activator is involved in the progression of thyroid cancer by remodelling the extracellular matrix [5,10]. Increases in transmembrane proteases such as aminopeptidase N (APN) and dipeptidylpeptidase IV (DPP IV) are more specific to thyroid carcinoma [11,12]. DPP IV activity is increased in some cancer types (e.g. thyroid cancer, prostate cancer, [13,14] and decreased or lost in others (e.g. melanoma, [15,16]). DPP IV regulates contact inhibition, cell cycle, morphological differentiation, tissue inhibitors of metalloproteinases, anchorage-dependent growth and E-cadherin of epithelial cancers [17]. A presumed role of DPP IV in cancer is in the induction of metabolic changes creating a favourable climate for the development of the tumor [17]. As DPP IV is occasionally expressed in thyrocytes of benign thyroid disorders [18] a link to proliferation has been suggested [11]. Increased APN expression is correlated with progression and metastasis in colorectal, pancreatic carcinoma and undifferentiated thyroid carcinoma [12,19,20].

Dipeptidyl peptidase II (DPP II, EC 3.4.14.2), a lysosomal protease ubiquitously expressed in many cells including normal thyrocytes of mammalian origin [21], is thought to play a role in the release of hormone from thyroglobulin [22].

Dipeptidyl peptidase IV (DPP IV, CD26, EC 3.4.14.5) is a trans-membrane type II glycoprotein with multifaceted function. As well as the integral membrane form, a soluble form is found in serum and semen. In contrast to thyroid follicle cells, most other types of human cell express DPP IV [23]. DPP IV is most up-regulated in papillary thyroid carcinoma [24,25] and apparently induced by RET/PTC mutations [26].

Aminopeptidase N (APN, aminopeptidase M, alanine aminopeptidase, CD13, EC 3.4.11.2), is expressed in anaplastic thyroid carcinoma cells but not in normal thyrocytes [12]. In porcine thyrocytes, by contrast, APN is a marker of the apical thyrocyte membrane [27,28].

To identify species with an identical pattern of protease activity compared to human thyrocytes, here we localized protease activities using synthetic substrates. The activities of DPP II, DPP IV and APN were studied in animal species used for investigating thyroid function, namely human, porcine, rat, mouse, bovine and ovine thyrocytes.

## Methods

### Tissue samples

Cadavers of rats (female, Sprague–Dawley) and mice (female, Balb/c), which had been used for other experiments, were obtained from the Institute of Pharmacology and the Institute of Anatomy, respectively. Porcine, bovine and ovine thyroid glands were obtained from the local slaughterhouse. Samples from human thyroid tissue were obtained from the surgical department of the University after informed consent of the patients. Animal procedures were performed according to the guidelines of the local authorities. All experiments on human subjects were conducted in accordance with the Helsinki Declaration of 1975.

For the localization of DPP IV and APN activities unfixed tissues were used. For the demonstration of DPP II 0.5 cm^3^ cubes of thyroid tissue were fixed in neutral buffered 4% formaldehyde solution with 30% sucrose for 24h at RT. After fixation, samples were rinsed for 24h at RT in distilled water containing 30% sucrose and 1% gum arabicum. Tissue samples were embedded in Tissue Tec (Miles) and deep-frozen in isopentane per-cooled with liquid nitrogen.

### Detection of protease activity

Protease activity in tissues and in cells was detected by cleavage of specific synthetic substrates. The synthetic substrate is bound to a tag, which together with the coupler yields a colored product, when released from the substrate.

Substrates coupled to 4-methoxy-2-naphthylamide (MNA, Bachem Ltd.) were used at a concentration of 0.5 mg/ml. Visualization of the reaction product was achieved through the presence of 1 mg/ml Fast Blue B (FFB, pure, tetrazotized Di-2-anisidine ZnCl_2_, Serva) in the reaction mixture.

10 μm cryostat sections were pre-treated with an ice-cold mixture of acetone and chloroform (1:1) for 5 min. Slides were air-dried for 30 min at RT prior to the incubation with the substrate solution.

The following substrates were used: Gly-Pro-MNA in 0.1 M PBS pH 7.0 for DPP IV, Ala-MNA in 0.1M PBS pH 7.0 for APN and Lys-Ala-MNA in 0.1 M cacodylate buffer pH 5.5 for DPP II [29]. Incubation time was 30 min for APN and DPP IV and 60 min for DPP II at 37°C. After washing in bi-distilled water slides were mounted with Kaiser's glycerol gelatine (Merck). Some sections were counterstained with hemalaun. For controls, the group-specific inhibitors (1 mM phenylmethanesulfonylfluoride and 1 mM diisopropylfluorophosphate, Sigma for DPP II and DPP IV and 10 mM 1,10-phenanthroline, Serva) were included in the incubation mixture.

## Physiological characterization of thyrocytes

### Cell culture

Primary culture of porcine thyrocytes was performed as described previously [30]. In brief, connective tissue was removed from thyroids of 10–20 pigs and thyroid glands were dissected into pieces of 0.5 - 1 cm^3^. The pieces were incubated with 1 l 0.5% dispase II (Roche) in Earle’s salt solution (Gibco) for 2h at 35°C. The incubation solution was constantly stirred and aliquots of 150 ml were taken and sieved through a tea sieve. The cell suspensions were diluted 1:3 with Earle’s solution and centrifuged (200 g for 7 min at 4°C). Cells were cultured in 6-well culture plates (Falcon®) at a density of 3x10^6^ cells/well in NCTC-135 medium supplemented with Ultroser G (3% v/v; Biosepra) and 1 μg/ml hydrocortisone and antibiotics. Human thyrocytes were also isolated from euthyroid goiters using the same protocol. 1 mU/ml porcine TSH (Sigma-Aldrich) was added to induce the formation of follicles. Cells were also cultured in the absence of TSH.

Cell number and cell viability were determined in an automatic mode based on the electrical sensing zone method (CASY Technology).

For the localization of protease activities, cells (1.5x10^6^) were seeded on cover slips placed at the bottom of 6-well plates. After 48 h of incubation, cover slips were either fixed in neutral buffered 4% formaldehyde solution with 30% sucrose for 10 min at RT, rinsed in PBS and infiltrated for 30 min at RT in distilled water containing 30% sucrose and 1% gum arabicum or placed immediately into an ice-cold mixture of acetone and chloroform (1:1) for 5 min and then stored at −20°C until assayed for protease detection (see above).

### Iodide uptake

For iodide uptake, 2.6 x10^5^ cells/well were plated in 48-well plates (Costar®) and treated with either 1.3 mU/ml TSH (Sigma) alone or TSH in combination with 2 mM thiamazole (Favistan®, Temmler Pharma) or 1 mM sodium perchlorate (99.9%, VWR International). Four kBq/well carrier-free Na^125^I (Amersham Biosciences) was added 6h prior to the measurement. Control cells were cultured in the absence of TSH. Cells were collected after 24h and 30 h of incubation and washed with a 48-well cell harvester (IH110, Inotech) with 1 μM NaI included in the washing solution. Filtermats (type 11731, Skatron) were transferred to counting tubes and measured (1480 automatic Gamma counter, Wallac).

The Dunnett test was used for statistical analysis. Results were considered statistically significant when p < 0.05. Mean ± SEM of n = 4 experiments.

### Ultrastructural analysis

Cells were cultured on gas-permeable hydrophilic polyfluoroethylene membranes (Petriperm, Heraeus) and fixed for 2h in 2.5% glutaraldehyde in 0.05 M cacodylate buffer pH 7.4 containing 2% sucrose, washed and post-fixed in 1% aqueous osmium tetroxide in 0.2 M buffer for 2h. Samples were dehydrated and embedded in Epon. Sections were cut, stained with saturated aqueous uranyl acetate (20 min) and lead citrate (5 min) and viewed with a LEO 912 OMEGA (Zeiss) transmission electron microscope.

## Results

### Protease activities in thyroid tissue

Because not all samples were collected at the same time, and the period between collection and freezing varied between 1h and 2.5h, time-dependent changes in the staining intensities were investigated over 4h in porcine thyroids. Despite a slight decrease of the staining intensity over this time, no loss of stained structures was observed. Perifollicular cells, which express all tested protease activities, served as controls that protease activities could be detected in the tissue.

**Figure 1:**
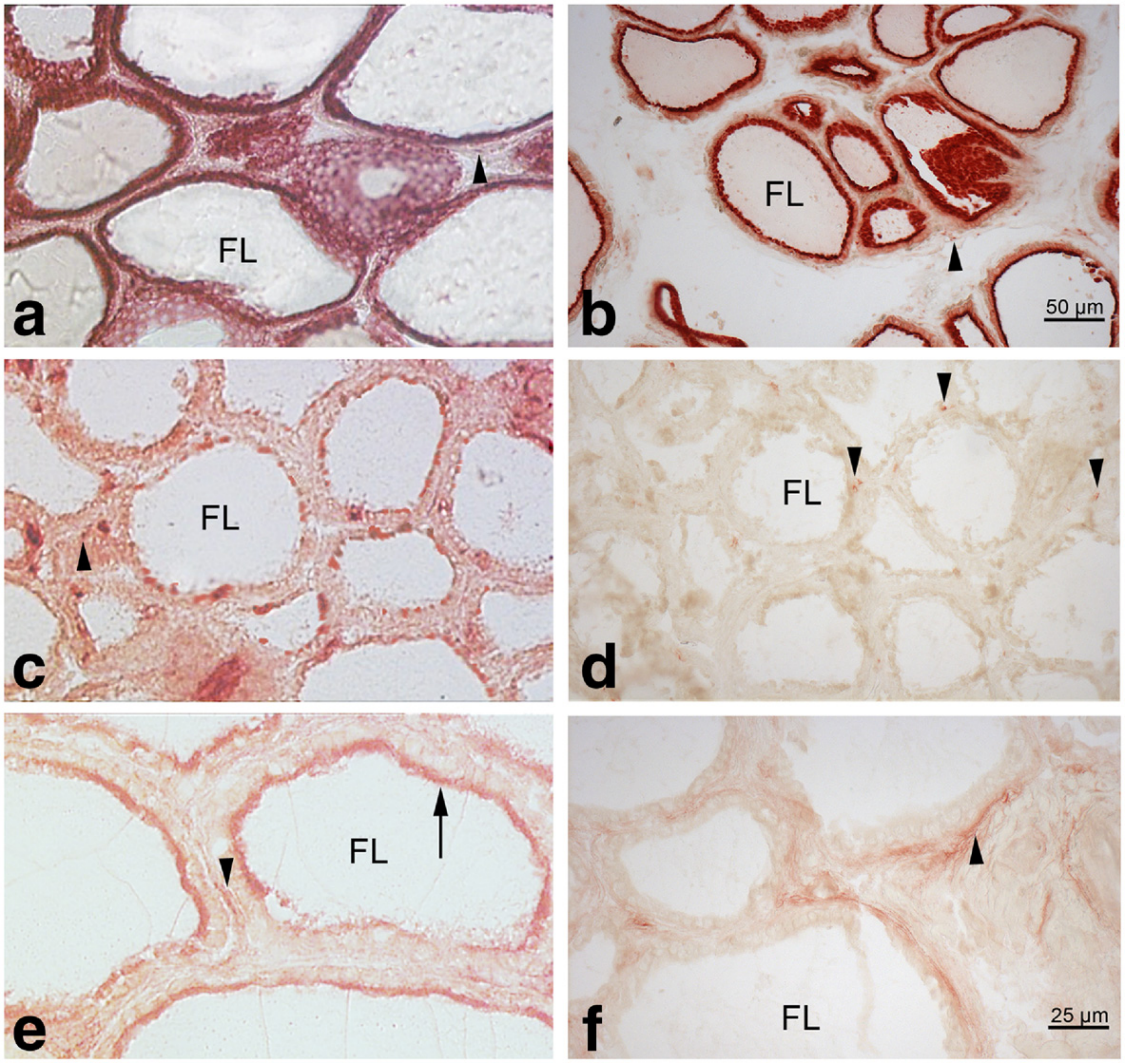
**Detection of protease activity with synthetic substrates by histochemistry (red) in porcine (a, c) and bovine (b, d) thyroid tissue.** Activities of perifollicular cells (endothelial cells, fibroblasts and C-cells) for the respective proteases are indicated by arrowheads. **a, b:** Activity of dipeptidyl peptidase II is seen intracellularly in thyrocytes of both species. c: In porcine thyroids activity of dipeptidyl peptidase IV is seen in some follicle cells. **d:** In bovine thyroids, follicle cells show no activity for dipeptidyl peptidase IV substrate. **e:** Activity of aminopeptidase N is seen at the apical part of the cells in porcine follicular thyrocytes (arrow), whereas in bovine thyroids **(f)** activity of aminopeptidase N is restricted to endothelial cells the perifollicular stroma below the follicular epithelium (arrowhead). FL: follicle lumen.

Activity of DPP II was detected in mouse, rat, human sheep, pig and cow thyrocytes (porcine and bovine thyroid shown, Figure 1 a, b). Activity of DPP IV and APN was absent in all these species (eg. bovine thyroid, Figure 1d) except porcine (Figure 1c). In all species, endothelial cells stained for APN activity and occasionally also for DPP IV activity. In porcine thyrocytes some, but not all, follicular thyrocytes displayed DPP IV activity (Figure 1c). Activity was localized in the cytoplasm and at the apical membrane.

Porcine thyrocytes also showed strong APN activity at the apical pole of the cell (Figure 1e). In addition to thyrocytes, also endothelial cells weakly expressed APN activity. In the other species studied, APN activity was restricted to endothelial cells in the peritumoral stroma (Figure 1f).

### Morphology, iodide uptake and protease activities in cultured thyrocytes

In human thyrocytes, only DPP II but no activities for APN and DPP IV were detected, suggesting that the isolation from the tissue did not cause prominent changes in the pattern of protease activities. To determine whether isolated cultured porcine thyrocytes also behaved similarly to thyrocytes in intact tissue, these cells were physiologically characterized.

**Figure 2:**
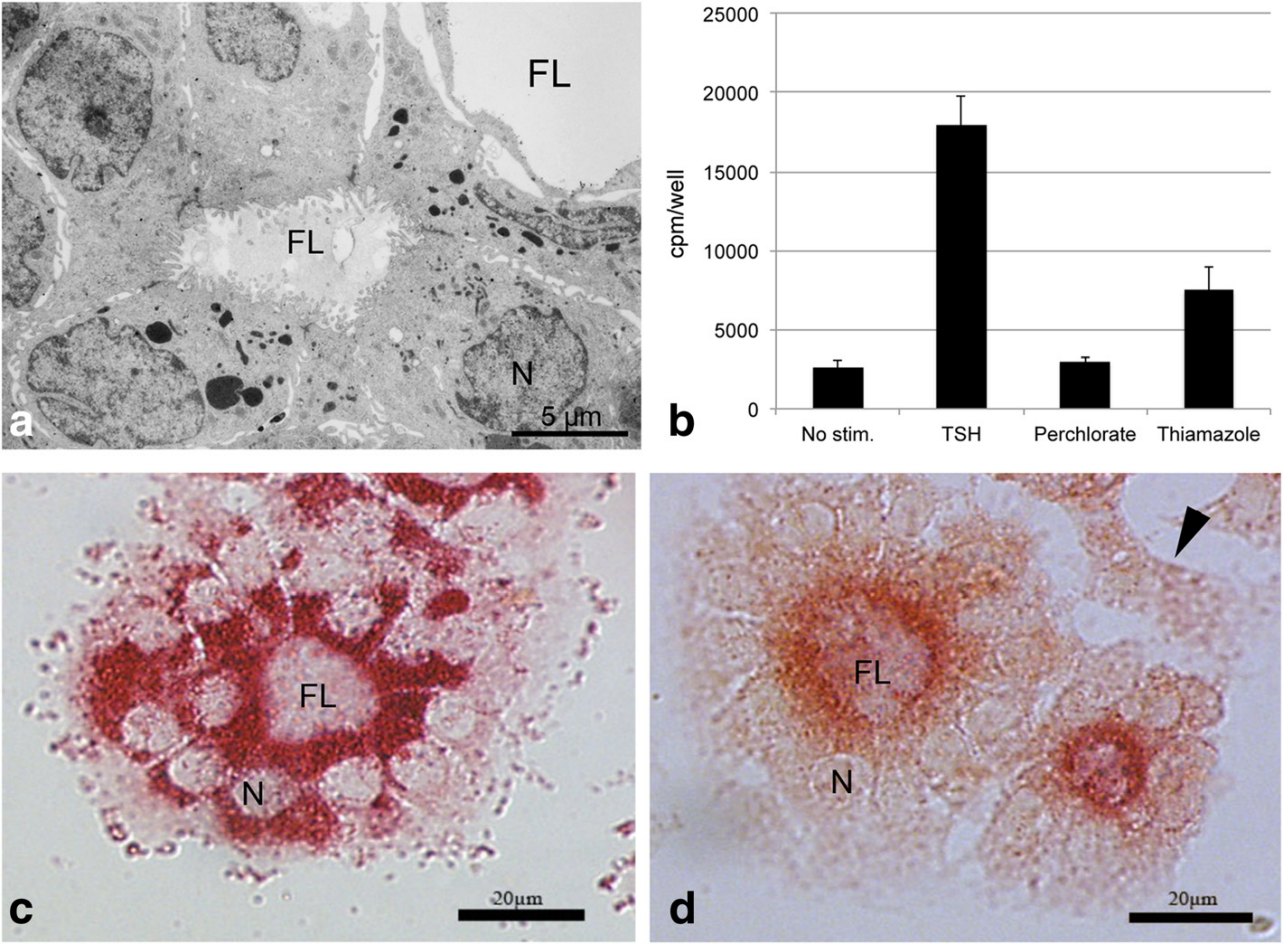
**Physiological behaviour of cultured porcine thyrocytes according to ultrastructure, iodide uptake and protease activity detected by synthetic substrate (red).a:** Porcine thyrocytes form follicles with formation of apical microvilli and intercellular tight junctions when stimulated with 1.3 mU/ml TSH for 30h. **b:** Upon stimulation with TSH, iodide uptake of thyrocytes is increased 6.8 times compared to unstimulated cells (mean ± SEM is shown). TSH-induced iodide uptake is inhibited by 1 mM perchlorate and significantly reduced upon exposure to TSH + 2 mM thiamazole (p < 0.05). **c:** Upon stimulation with TSH for 30h, DPP II activity is seen in all cells, whereas activity of APN at the plasma membrane in seen only in thyrocytes integrated in follicles but not in isolated cells (d, arrowhead). N: nucleus, FL: follicular lumen.

Porcine thyrocytes formed functional follicles with characteristic thyrocyte morphology and with a stable preserved polarity in the presence of TSH (right-side-right follicles, Figure 2a). These follicles showed microvilli at the apical surface and tight junctions between the cells, but no basement membrane formed at the basal pole of the cells.

Upon stimulation with TSH, iodide uptake was increased by a factor of 6.8 relative to unstimulated controls (Figure 2b). This uptake was inhibited by 1mM perchlorate. Despite being an inhibitor of iodine organification, not of iodide uptake, thiamazole also significantly decreased iodide-uptake.

Activities for all enzymes detected in intact tissues were also demonstrated in primary cultures of porcine thyrocytes when cultured in the presence of TSH. Intracellular localization of DPP II was seen in all cells (Figure 2c), but only thyrocytes integrated into follicles showed localization of APN at the plasma membrane (Figure 2d). Compared to APN, DPP IV activity was very weak. When cultured in the absence of TSH in porcine thyrocytes only DPP II could be detected (data not shown), whereas the activities of APN and DPP IV were below the detection threshold. In human thyrocytes, only DPP II activity, but not APN and DPP IV was detected.

## Discussion

We report differences in the activities of two membrane-associated proteases, DPP IV and APN, between thyrocytes from human and animal sources. These differences may reflect profound differences in the regulation of some proteases between human and porcine thyrocytes because the expression of DPP IV and APN is linked to transformation of human thyrocytes.

Thyrocytes from animals play an important role in the study of physiological processes because inter-individual variations in animals usually are lower than in humans. Inter-individual variations in protein expression, iodide uptake, proliferation and other physiological reactions are more pronounced in normal human thyroid tissue samples and isolated human thyrocytes than in porcine ones [31]. Causes of different physiological reactions among individuals include genetic factors and environmental factors (dietary iodine, smoking, infections, etc.). Due to these limitations, porcine, bovine, ovine and canine thyrocytes are common substitutes for normal human cells because only animal thyrocyte lines able to form follicles and synthesize thyroid hormones are available [1,32]. Despite general similarities in morphology, synthesis of thyroid hormone and the reaction to TSH, several differences between the species, including molecular differences in proteins, in expression and in reaction to growth factors, have been identified [2,33-36].

In our study, lysosomal protease activity of DPP II was strongly expressed in thyrocytes of all species. This lack of interspecies differences was also reported in another study on the expression pattern of the lysosomal proteases cathepsin B and elastase in the placenta of mice, rats, guinea pigs and marmosets [37]. In contrast, we saw expression of DPP IV and APN only in porcine thyrocytes but not in thyrocytes from other species. In human thyroid glands, consistent with previous studies, thyrocytes lacked both enzyme activities and only endothelial cells showed reactivity for DPP IV [38]. Pronounced interspecies variations in the expression of the membrane-associated proteases were also reported by Gossrau and Graf, who investigated cellular expression of γ-glutamyltranspeptidase, aminopeptidase A, APN and DPP IV activities [37].

The observed differences in protease activities persisted in cultured porcine cells when cultured in the presence of TSH. As the membrane-associated proteases DPP IV and APN localize to the apical membrane, they are only expressed when follicles are formed. This indicates that, contrary to human thyrocytes, they are markers of differentiation, not de-differentiation. Expression of APN in porcine thyrocytes has also been reported by Feracci et al. [27]. Because of these observed differences, porcine thyrocytes are not suitable models for studies on the regulation of membrane-protease in human thyrocytes.

The determination of actual protease activity in this study, instead of merely detecting protein or mRNA, allows a direct assessment of relevant functional activity. Protease activity is regulated at different levels, including alternative splicing, compartmentalization, pH, aggregation of subunits, inhibitors etc. [39,40] and often a correlation between mRNA expression and protease activity is lacking [41]. Nevertheless, absence of mRNA does indicate absence of the protein and is, therefore, useful because a lack of cross-reactivity of the available antibodies hinders interspecies comparisons.

One problem in the evaluation of protease activity by synthetic substrates may be the lack of specificity of these peptides. Although different proteases degrade similar substrates in vivo, the choice of the fixation, evaluation of the staining by microscopy as well as the inclusion of appropriate inhibitors makes false positive results in this study highly unlikely. Peptides with proline in the penultimate position at the amine terminus are only cleaved by DPP IV and its homologues [42]. APN selectively cleaves peptides with alanine in the penultimate position. Activities of DPP IV and APN are inhibited almost completely by inclusion of diisopropyl fluorophosphate and 1,l0-phenanthroline, respectively [43], showing that under the conditions used, the staining is specific. Differentiation between proteases with similar substrate specificity and catalytic centers, for instance DPP II and DPP IV, can be achieved by using the appropriate fixation protocols [44].

We also showed here that differences between porcine and human thyrocytes are not restricted to the expression of protease activities. Although porcine thyrocytes re-organized into follicle-like structures similar to those seen in human, the TSH-induced increase in iodide uptake was slightly smaller than reported for human cells (7–10 times,[45,46]). More importantly, the reaction to thiamazole differed between porcine and human thyrocytes. Whereas these inhibitors of iodide organification have no effect on iodide uptake in cultured human thyrocytes [47], they depressed iodide uptake in our study (porcine thyrocytes) as well as in studies on canine thyrocytes [48,49].

## Conclusion

The presented data show that expression of membrane-associated proteases in thyrocytes is subject to inter-species variations. Although thyrocytes from animals are useful tools for the investigation of human thyrocytes, for studying protease changes porcine thyrocytes appear to be less suited than thyrocytes from other species.

## Abbreviations

DPP IV: Dipeptidyl peptidase IV; APN: Aminopeptidase N; DPP II: Dipeptidyl peptidase II.

## Competing interests

The authors declare that there are no competing financial interests.

## Authors’ contributions

EF, RW: interpretation of data and writing of manuscript, EM: generation and interpretation of data. All authors read and approved the final manuscript.
